# A New Role for the HTLV-1 p8 Protein: Increasing Intercellular Conduits and Viral Cell-to-Cell Transmission

**DOI:** 10.3390/v3030254

**Published:** 2011-03-09

**Authors:** Marine Malbec, Ferdinand Roesch, Olivier Schwartz

**Affiliations:** Virus and Immunity Unit, Institut Pasteur, URA CNRS 3015, 28 rue du Dr. Roux, 75724 Paris, France

**Keywords:** HTLV-1, HIV-1, cell-to-cell transfer, virological synapses, cellular conduits, p8, p12, Nef

## Abstract

Retroviruses like HIV-1 and HTLV-1 can be transmitted efficiently by direct contact between infected and target cells. For HIV-1, various modes of cell-to-cell transfer have been reported, including virological synapses, polysynapses, filopodial bridges, and nanotube-like structures. So far, only synapses and biofilms have been described for HTLV-1 transmission. Recently, Van Prooyen *et al.* [1] identified an additional mode of HTLV-1 transmission through cellular conduits induced by the viral accessory protein p8.

Human T-cell leukemia virus type 1 (HTLV-1) is the causative agent of adult T-cell leukemia/lymphoma (ATLL), a rare but aggressive T-cell lymphoproliferation. Infection by HTLV-1 is also associated with tropical spastic parapesis/HTLV-1-associated myelopathy (TSP/HAM), a neurological disorder. This retrovirus is barely secreted from infected cells, but is efficiently transmitted by cell/cell contacts such as virological synapses (VS) [2,3] or polysynapses (PS) [1], and propagates by clonal expansion of infected T cells. Like many other retroviruses, HTLV-1 encodes regulatory (HBZ, Tax, Rex) and accessory (p12, p13, p30) proteins. It has been previously shown that p12, which is encoded by the *orf-I* gene, can be cleaved in the endoplasmic reticulum (ER) to generate p8, a membrane-associated protein [4]. Many different activities have been reported for p8 and p12, but the specific contributions of these two proteins are still unclear. A recent article by the team of Genoveffa Franchini provides novel insights into the role of these proteins and reports that p8 increases HTLV-1 cell-to-cell transmission through the formation of cellular conduits [1].

To investigate the role of p12 and p8 in HTLV-1 transmission, Van Prooyen *et al*. [1] transfected cells with WT, p8/p12-deleted HTLV-1, or with plasmids expressing p8 or an uncleavable form of p12. They show that expression of p8, but not that of the uncleavable variant of p12, rescues p8/p12-deleted virus infectivity defect. p8 is recruited at the cell membrane and colocalizes with lymphocyte function-associated antigen-1 (LFA-1). Even if p8 does not affect LFA-1 surface levels, it increases its clustering at the cell surface which results in an increase of T-cell adhesion, conjugate formation and HTLV-1 transmission. Coculture experiments demonstrated that p8 increases the number and the length of cellular conduits. Furthemore, p8 overexpression in a chronically HTLV-1 infected cell line increases polysynapses formation and viral transmission. In addition to Gag, p8 is also transferred to neighboring cells through these conduits. The authors conclude that HTLV-1 can be transmitted upon contact with the target cell, via the virological synapse, and through cellular conduits. They propose a model in which p8 would enhance viral transfer, and at the same time invade target cells to protect them from immune recognition (Figure 1).

This work suggests the existence of a new mode of HTLV-1 transmission mediated by p8, and reconciles some contradictory observations concerning the p8/p12 proteins. However, the molecular mechanism by which p8 promotes HTLV-1 transmission remains unknown. Especially, it remains unclear whether p8-mediated increase of viral transmission is mediated by (i) cellular conduits, as suggested by the authors (ii) transfer through virological synapses and polysynapses (iii) syncytia, as proposed by Taylor *et al.* [5] or (iv) through “viral biofilms” [6]. Moreover, this study relies mostly on protein overexpression in a cell line model. It will be important to confirm the relevance of these results in primary T cells, using viruses deleted for p8 or p12 only, rather than complementing p12-deleted viruses with overexpressed p8 or p12. Finally, it has been previously demonstrated that p8 is present in lipid rafts and colocalizes with the immune synapse (IS), where it down-regulates expression of several proteins such as Major Histocompatibility Complex type I (MHC-I) and T cell receptor (TCR). Thus, it would be of great interest to determine if p8 is also present at the site of the VS and influences viral transfer.

Like HTLV-1, Human immunodeficiency virus type 1 (HIV-1) is transferred directly from cell to cell, using filopodes, nanotubes, VS and PS. Interestingly, the features of p8 are highly reminiscent of those of the HIV-1 Nef protein (Table 1).

HTLV-1 and HIV-1 use viral synapses to promote efficient transmission. They both encode one accessory protein present in the lipid rafts, targeting similar cellular functions, such as MHC-I and TCR down-regulation, and impairment of immune synapse formation. Both Nef and p8 induce the formation of membrane extensions, which may allow them to be transferred to target cells. Nef, for instance induces the formation of intercellular conduits bridging infected macrophages to bystander B lymphocytes (LB). Nef is then transferred to LB and inhibits IgG2 and IgA class switching [22]. Nef also promotes the formation of filopodia [18], but probably not that of nanotubes, an event independent of HIV infection [23]. In the present work, the authors show that p8 increases the number and length of cellular conduits and is responsible for a four-fold increase of HTLV-1 transmission. Therefore, it is tempting to speculate that Nef may have similar effects on HIV-1 cell-to-cell transmission. However, a recent report indicates that this is not the case [17].

Nef and p8/p12 appear to be dispensable for *in vitro* propagation. In contrast, viruses deleted for these proteins replicate less efficiently in non-human primates [9,10]. The *in vivo* relevance of the results described by Van Prooyen *et al.* [1] remains thus to be established. For instance, it has been proven difficult to detect p8 at the protein level in HTLV-1 infected cells, and Fukumoto *et al.* reported a predominance of alleles encoding uncleavable forms of p12 in a cohort of HTLV-1-infected patients [4]. More work is therefore required to confirm the importance of p8 in viral transmission *in vivo*.

Overall, this work identifies a new mode of HTLV-1 transmission using cellular conduits induced by the viral protein p8. To gain further insight into HTLV-1 transmission and pathogenesis, it will be worth better characterizing the specific functions of p8 and p12 *in vitro* and *in vivo* and identifying the cellular proteins involved in this new mechanism. Since HIV-1 Nef and HTLV-1 p8 share common features, it will be of interest to determine if these two proteins interact with the same host proteins to mediate their effects on viral replication.

## Figures and Tables

**Figure 1.**
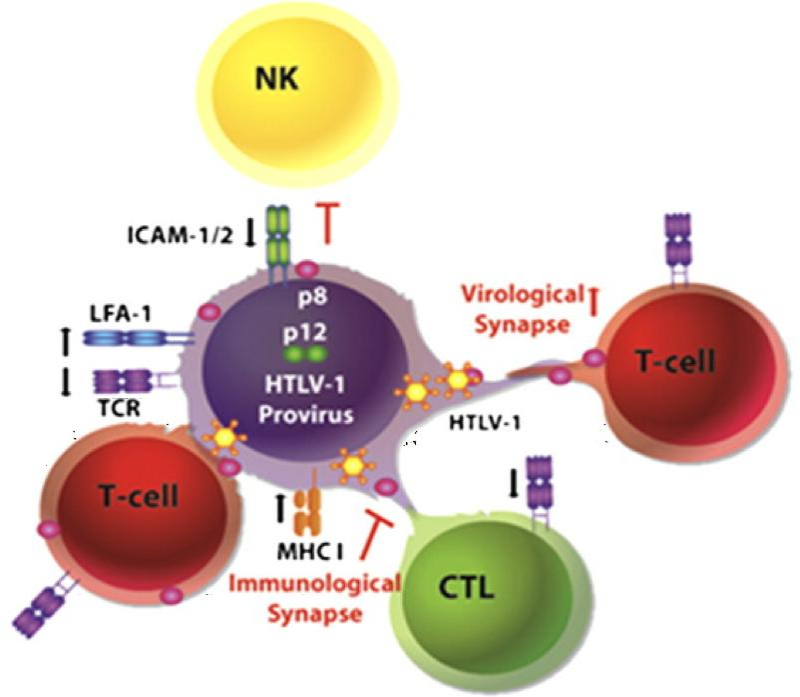
Hypothetical model for p8 and p12 action (from [1]). In infected cells, p8 decreases T cell receptor (TCR) proximal signaling, major histocompatibility complex type I (MHC-I) expression at the cell surface, and increases lymphocyte function-associated antigen-1 (LFA-1) clustering. These events result in low viral production, evasion from immune recognition by NK cells and CD8^+^ T cells (CTL) and recruitment of non-infected cells, respectively. p8 induces the formation of cellular conduits, allowing rapid transfer of the virus and of p8 itself into target cells. In newly infected cells, p8 promotes anergy and contributes further to immune evasion. This model provides clues for understanding the mechanism of HTLV-1 persistence in the infected host.

**Table 1.** Comparison of HIV-1 Nef and HTLV-1 p8/12 main functions.

**Functions**	**Nef**	**p8/p12**
Increase of infectivity *in vitro*	+ [7]	+ [8]
Required for infection *in vivo*	+ [9]	+ [10]
TCR down-regulation	+ [11]	+ [12]
MHC-I down-regulation	+ [13]	+ [14]
Increase of IL-2 production	+ [15]	+ [16]
Immune synapse modulation	+ [11]	+ [4]
Increase of viral cell-to-cell transmission	+/− [17]	+ [1]
Induction of membrane extensions	+ [18]	+ [1]
Syncytia formation	↓[19]	↑ [5]
Polysynapse formation	?	+ [1]
ICAM 1-2 down-regulation	?	+ [20]
LFA-1 clustering	?	+ [21]
